# Supplementary material for: Super-resolution microscopy reveals functional organization of dopamine transporters into cholesterol and neuronal activity-dependent nanodomains
Source: Nat Commun. 2017 Sep 29;8:740. doi: 10.1038/s41467-017-00790-3 (PMC5622129; doi:10.1038/s41467-017-00790-3)
Supplement: Supplementary file 1 — Supplementary information Supplementary figures [file 41467_2017_790_MOESM1_ESM.pdf]

**Description of Supplementary Files**

File Name: Supplementary Information

Description: Supplementary figures

File Name: Peer Review File

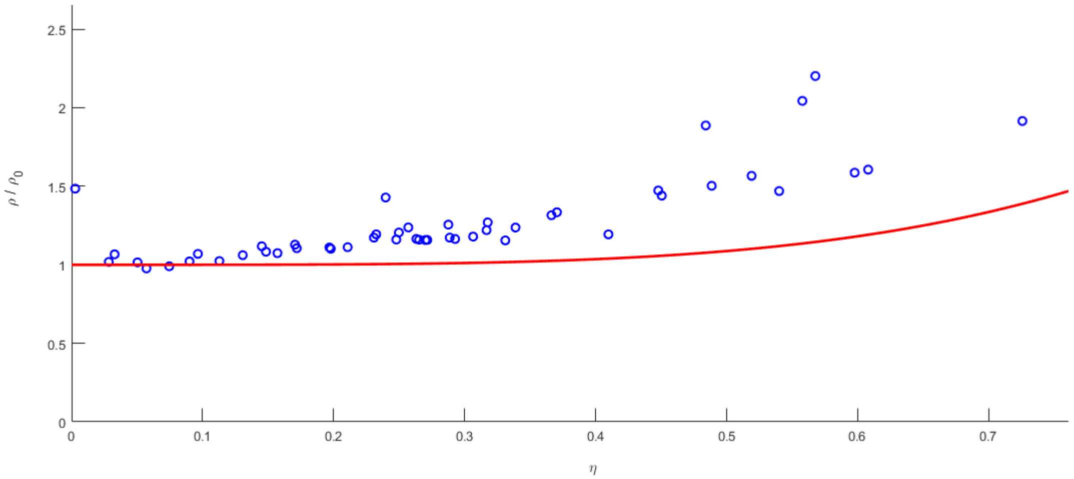


**Supplementary Figure 1**. Dronpa-DAT forms clusters in the plasma membrane of CAD cells as demonstrated by an analysis method from Baumgart et al. (**a**) The normalized average density of localization present in clusters ($\rho/\rho_0$) was compared to the relative area of the ROI covered by a cluster mask ($\eta$), with each data point being a 5 µm by 5 µm ROI from a CAD cell expressing Dronpa-DAT and imaged with PALM. The red line indicates a reference curve from a random distribution.

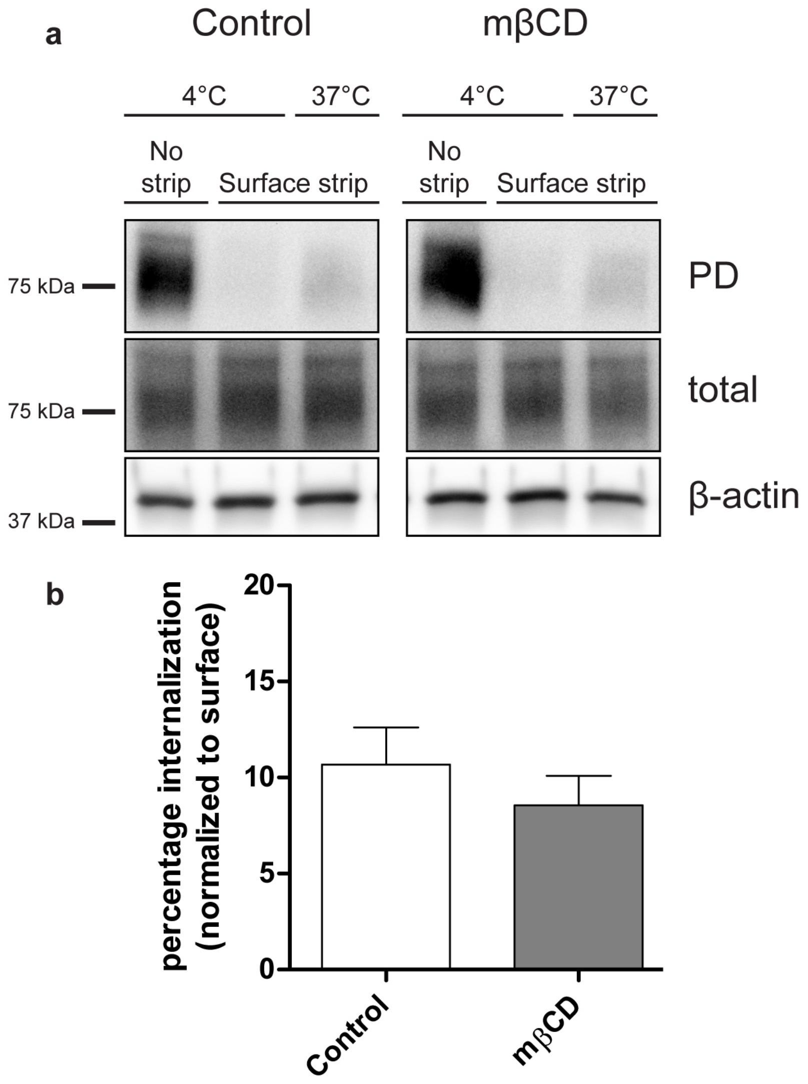


**Supplementary Figure 2**. Cholesterol depletion does not affect DAT internalization in CAD cells. Reversible biotinylation assay on CAD cells assessing the internalization of DAT over 30 min at 37°C without or with cholesterol depletion with mβCD (**a**) *Top panels*; western blotting for biotinylated, pulled-down DAT (PD) (control, mβCD). Lanes 1 and 2 from left (4 °C) show total surface immunosignal (no stripping with MesNa) and after stripping (+MesNa). Lane 3 shows internalized DAT (37 °C). Middle panels, western blotting for total DAT; Lower panels; western blotting for actin. (**b**) Quantifications of internalized DAT minus the stripping control as a percentage of total surface DAT (mean ± s.e.m. not significant according to a an unpaired, two-tailed t-test, control: n = 4, mβCD: n = 5.

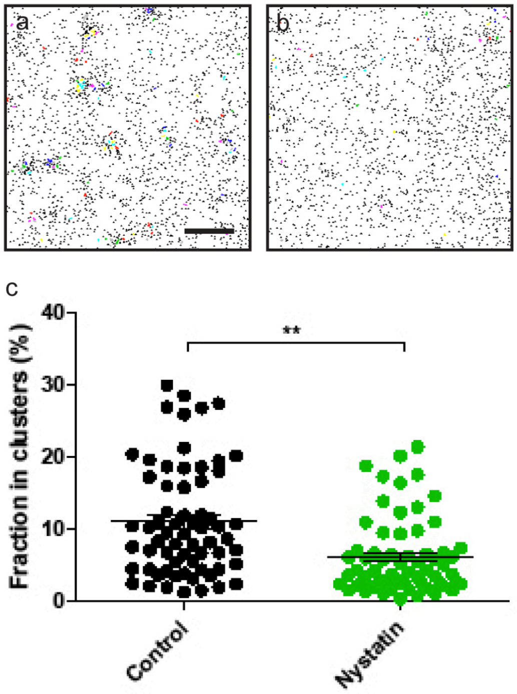


**Supplementary Figure 3.** Cholesterol sequestration by nystatin decreases DAT clustering in CAD cells. CAD cells transiently expressing Dronpa-DAT were treated for 20 min treatment at 37°C with vehicle or 10 µg $ml^{-1}$ nystatin prior to PALM imaging. (**a, b**) DBSCAN-based cluster maps of Dronpa-DAT distribution in CAD cells without (**a**) or with nystatin (**b**). Clustered localizations are shown by color-coding with non-clustered localizations in grey. (**c**) Clustering of Dronpa-DAT given as the fraction of localizations in clusters in % (mean ± s.e.m.,**$p<0.01$, unpaired, two-tailed t-test). Data are based on 129 cells from 3 independent experiments. Scale bar 1 µm

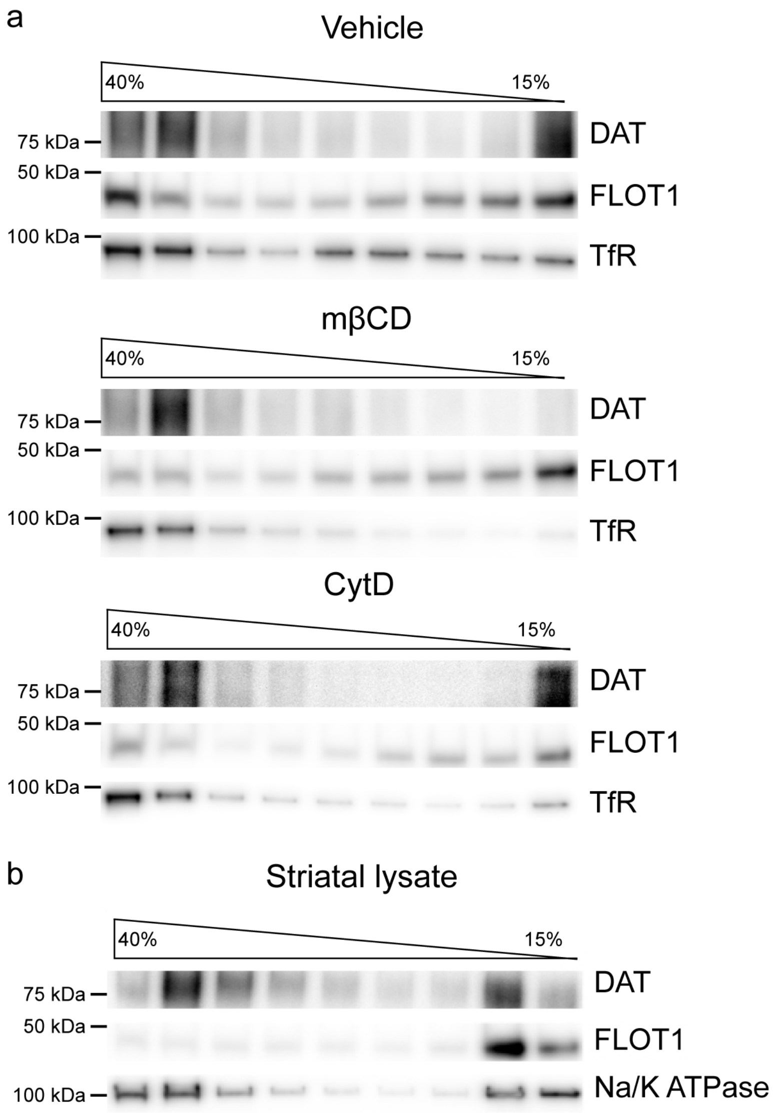


**Supplementary Figure 4**. DAT segregates into detergent-resistant low buoyancy membrane fractions in a cholesterol-dependent manner. (**a**,**b**) Sucrose-based density gradient fractionation of DAT in lysates of CAD cells and mouse striatum. Cells or mice striatal tissue were lysed using 1% Brij58 and mixed with 80% sucrose in a continuous sucrose gradient from 40-15%. Subsequently the sucrose gradient was centrifugated for 18 h at 100,000 x g and divided into ten fractions. The fractions were analyzed by western blotting using specific antibodies for indicated proteins. (**a**) Vehicle treated cells transiently expressing the transferrin receptor (TfR) and DAT. DAT segregates both into high-buoyancy fractions together with TfR and into low-buoyancy fractions together with the (endogenous) membrane-raft marker, flotillin-1 (FLOT1). Cholesterol depletion with 5 mM methyl-β-cyclodextrin (mβCD) but not actin depolymerization with 5 μg $ml^{-1}$ cytochalasin D (CytD) disrupted segregation of DAT into low-buoyancy fraction. Representative cropped western blots for entire membranes developed for horseradish peroxidase, see Supplementary Figure 8. (**b**) Endogenous DAT also segregates into both high-buoyancy fractions together with the $Na^+/K^+$ ATPase and into low-buoyancy fractions together with flotillin-1 (FLOT1). Immunoblots shown are representative of three experiments with similar results.

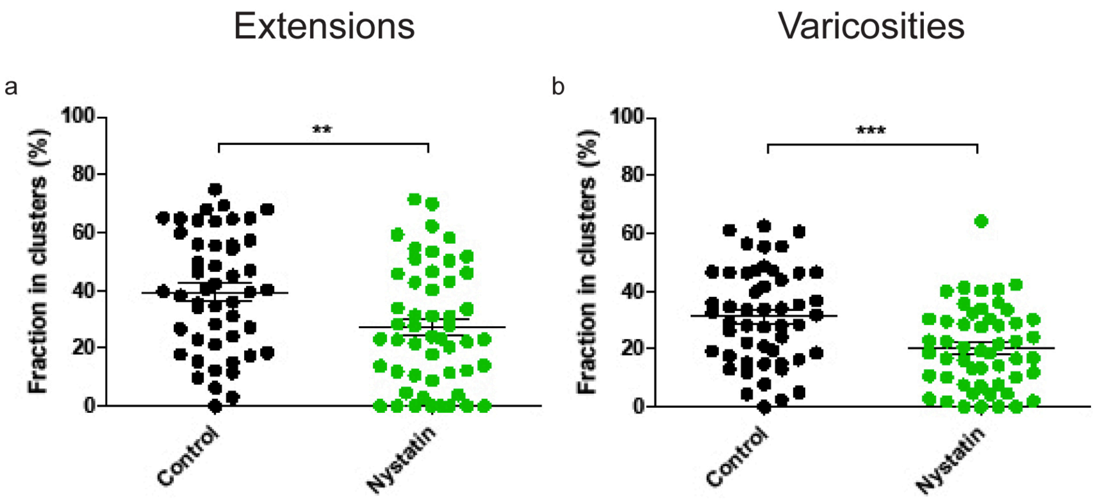


**Supplementary Figure 5.** Cholesterol sequestration by nystatin decreases DAT clustering in dopaminergic neurons. Cultered dopaminergic were treated for 20 min at 37°C with vehicle or 10 µg $ml^{-1}$ nystatin prior to fixation and staining for DAT using anti-DAT antibody and Alexa405-Alexa647-conjugated secondary antibody. The resulting STORM images were analyzed by DBSCAN. (**a, b**) Clustering of endogenous DAT as assessed by DBSCAN in neuronal extensions and varicosities given as the fraction of localizations in clusters in % (means ± s.e.m., **p<0.01, ***p<0.001, unpaired, two-tailed t-test). Data are based on 99 cells from 3 independent experiments.

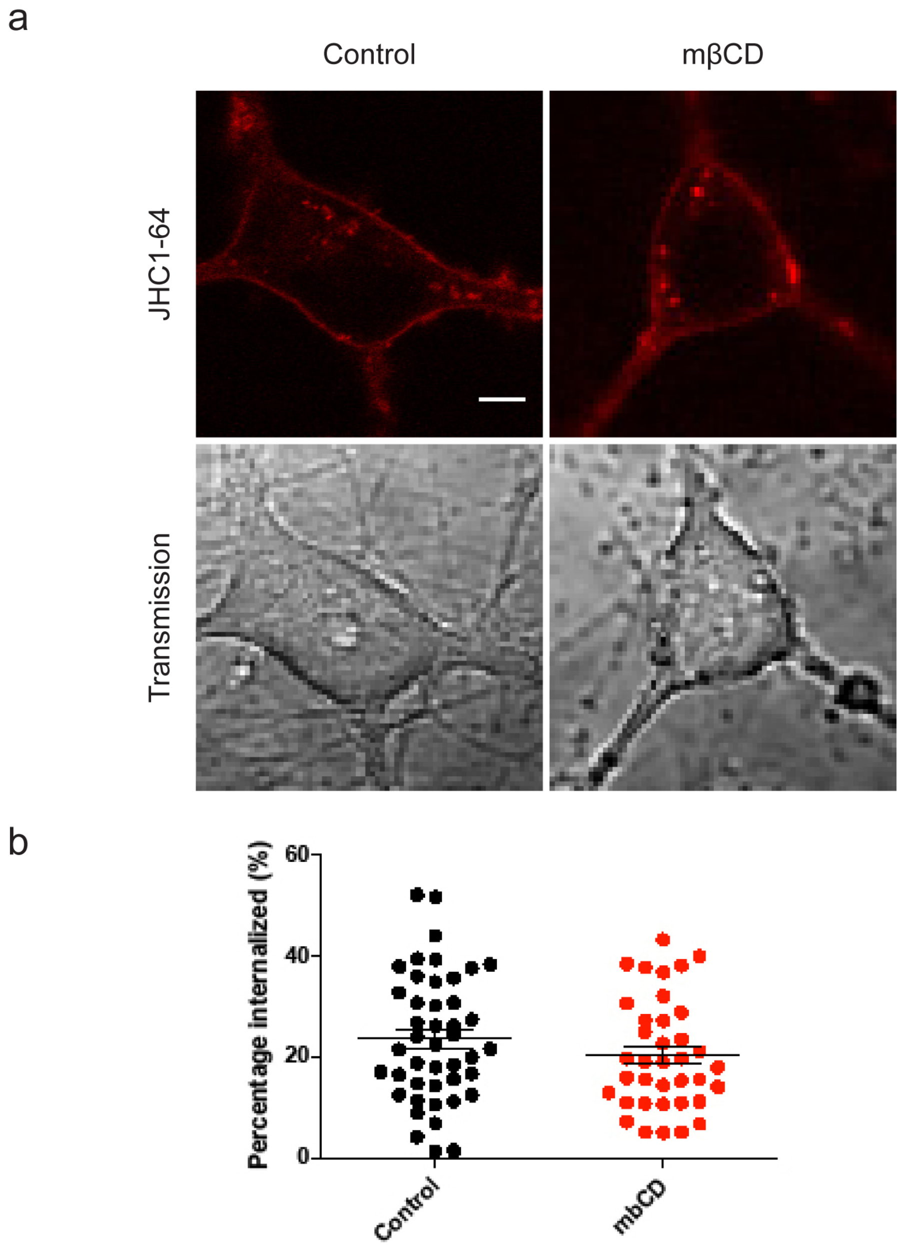


**Supplementary Figure 6**. Cholesterol removal does not influence the internalization DAT in cultured dopaminergic neurons. (**a**) *Top panel*, Confocal images of representative live dopaminergic neurons showing internalization of DAT labeled with the fluorescent cocaine-analogue JHC 1-64. The neurons were labeled for 20 min with 20 nM JHC1-64 at 4°C before incubation for 30 min at 37°C with vehicle (control) or 5 mM m $\beta$ CD followed by further 30 min incubation at 37°C. *Bottom panel*, transmission images of the cells shown in the *Top panel*. (**b**) Quantification of internalized JHC 1-64 – labeled DAT after 1 h at 37°C expressed as the fraction of intracellular JHC1-64 signal relative to the cell surface (Mean±s.e.m.; not significant according to an unpaired, two-tailed t-test). Data are based on 37-44 cells from 3 independent experiments. Scale bar 500 nm.

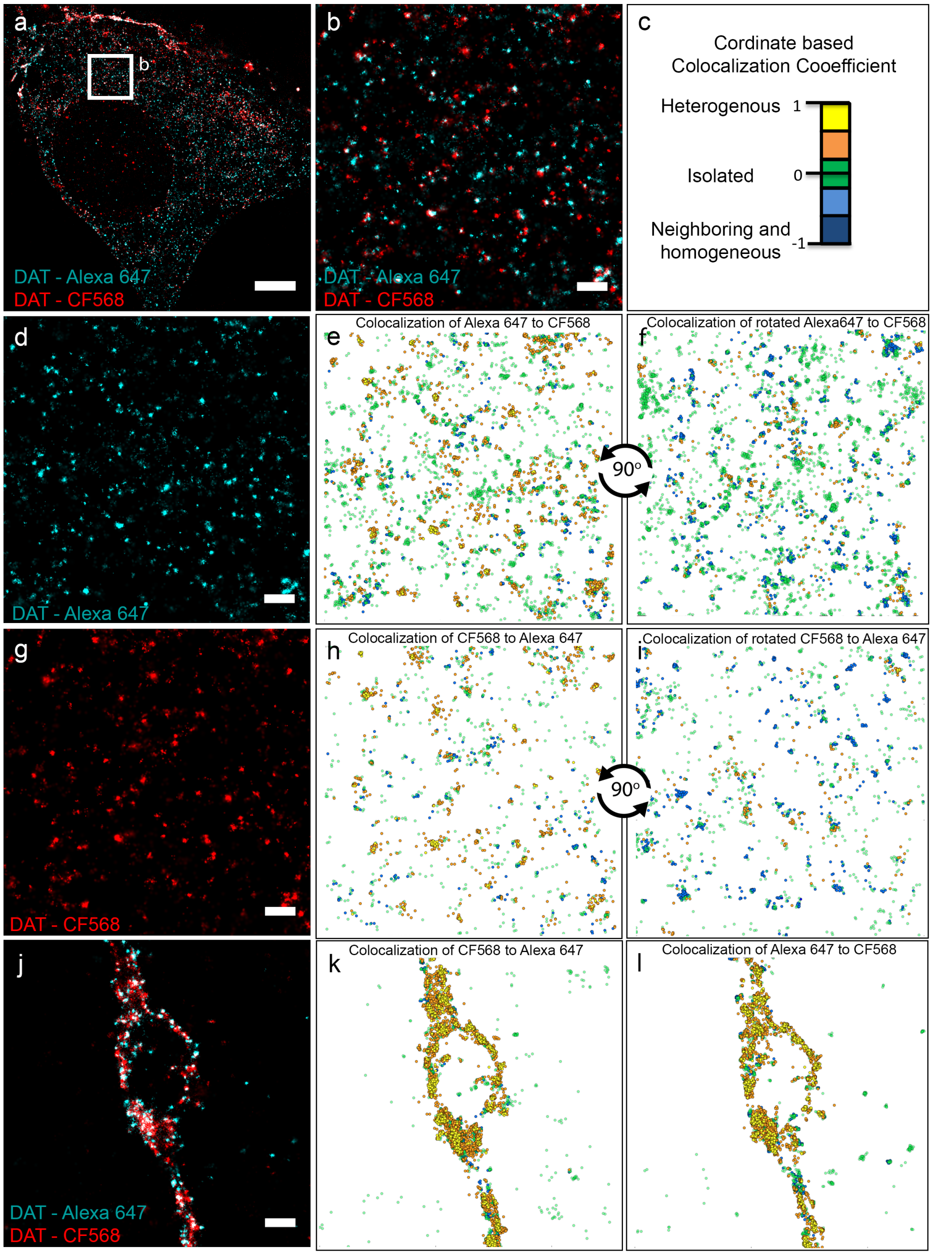


**Supplementary Figure 7**. Dual-color dSTORM images of DAT, colabeled with two spectrally separate fluorophore-conjugated secondary antibodies, identify similar protein distributions on dopaminergic neurons. (**a**) Cross-section views through the cell body of a dopaminergic neuron showing the distribution of DAT with the two spectrally separate labels by dSTORM imaging. (**b**) Enlarged view of the dSTORM image corresponding to the boxed region in **a**. (**c**) Color code for colocalization identification used in images **e,f,h,i**. (**d**) Localizations detected from Alexa 647 in image **b**. (**e**) Colocalization of Alexa647 localizations to CF568 localizations. (**f)** Colocalization of Alexa647 localizations after a $90^0$ rotation to CF568 localizations. (**g**) Localizations detected from CF568 in image **b**. (**h**) Colocalization of CF568 localizations to Alexa647 localizations. (**i**) Colocalization of CF568 localizations after a $90^0$ rotation to Alexa647 localizations. (**j**) Dual-color dSTORM image for DAT on a varicosity of a dopaminergic neuronal process. (**k**) Colocalization of CF568 localizations to Alexa647 localizations from image **j**. (**l**) Colocalization of Alexa647 localizations to CF568 localizations from image **j**. Scale bars **a** 4 µm, **b,d,g,j** 0.5 µm.

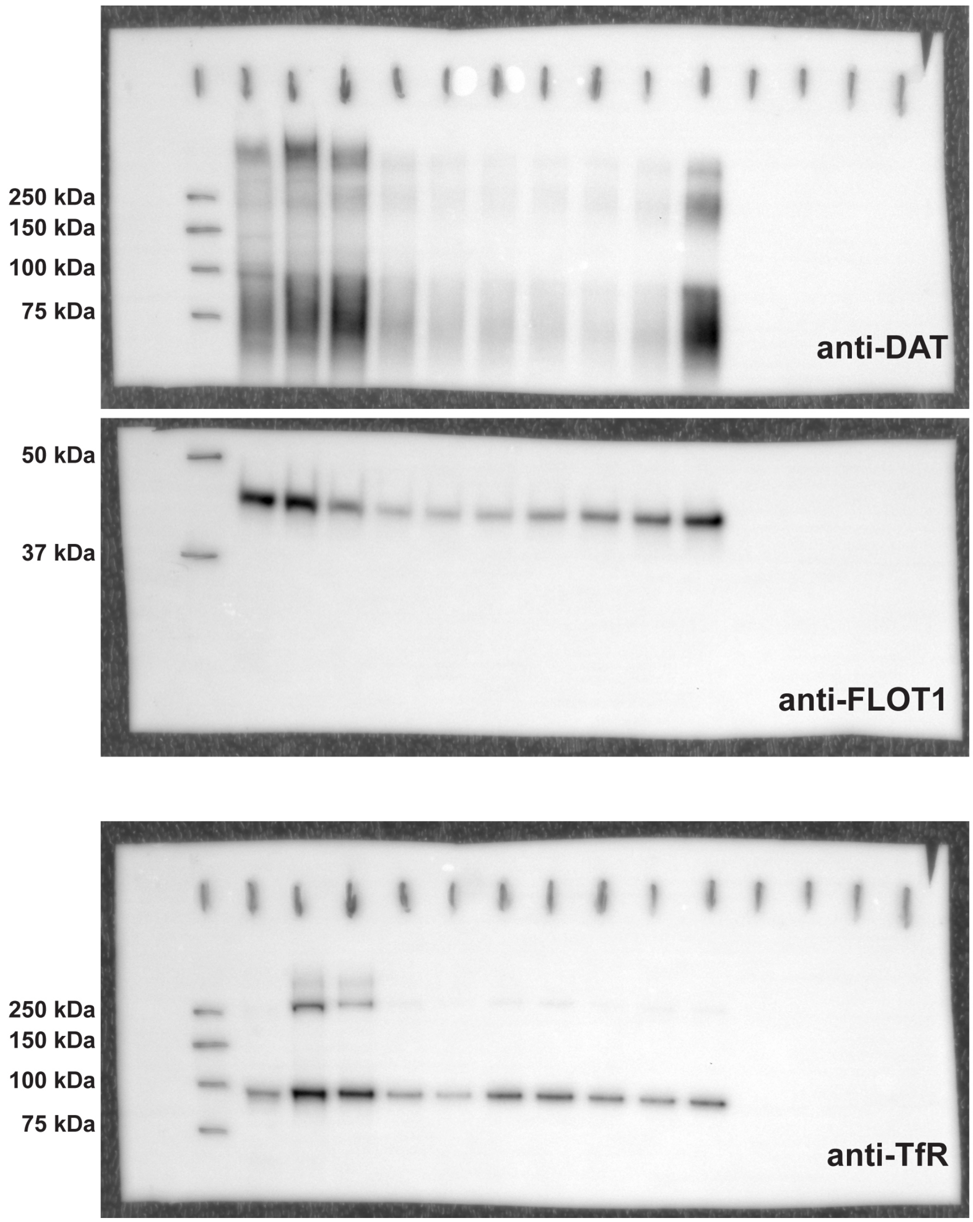


**Supplementary Figure 8.** Scan of uncropped western blot membranes of sucrose gradients samples developed for horseradish peroxidase. Sucrose gradient samples were loaded to an 'any KD', precast gel and transferred on to a PVDF membrane. The membrane was cut in two and developed for DAT using rat anti-DAT antibody, flotillin-1 (FLOT1) using rabit anti-FLOT1 antibody, and the transferrin receptor (TfR) using mouse anti-TfR antibody. The signal was amplified with HRP-conjugated secondary antibody and developed with ECL prime western blotting reagent.
